# The aggressive surgical treatment and outcome of a colon cancer patient with COVID-19 in Wuhan, China

**DOI:** 10.1186/s12876-020-01411-y

**Published:** 2020-08-14

**Authors:** Jinbo Gao, Ming Yang, Lian Liu, Shuang Guo, Yongfeng Li, Chao Cheng

**Affiliations:** 1 Department of Gastrointestinal Surgery, Union Hospital, Tongji Medical College, Huazhong University of Science and Technology, 1277 Jiefang Avenue, Wuhan, Hubei China; 2 Department of Pathology, Union Hospital, Tongji Medical College, Huazhong University of Science and Technology, Wuhan, Hubei China; 3 Cancer Center, Union Hospital, Tongji Medical College, Huazhong University of Science and Technology, Wuhan, Hubei China

**Keywords:** Colon Cancer, COVID-19, SARS-CoV-2, Pathology, Case report

## Abstract

**Background:**

Cancer patients are at increased risk of novel coronavirus disease 2019 (COVID-19). Currently, surgeries for cancer patients with COVID-19 are generally suggested to be properly delayed.

**Case presentation:**

We presented a 69-year-old Chinese female colon cancer patient with COVID-19, the first case accepted the surgical treatment during the pandemic in China. The patient developed a fever on January 28, 2020. After treatments with Ceftriaxone and Abidol, her fever was not moderated yet. A repeat chest computed tomography (CT) scan showed significantly exacerbated infectious lesions with a positive result for severe acute respiratory syndrome coronavirus 2 (SARS-CoV-2) nucleic acid. An abdomen CT scan indicated the tumor of ascending colon with local wrapped changes. She was diagnosed with ‘Severe novel coronavirus pneumonia’ and ‘Incomplete bowel obstruction: Colon cancer?’. After actively anti-inflammatory and anti-viral therapies, a right colectomy with lymph node dissection was performed on March 11, followed by a pathological examination. The patient successfully recovered from COVID-19 pneumonia and incomplete bowel obstruction after surgery without any postoperative related complications and was discharged on the 9th day after operation. Significant degeneration, necrosis and slough of focal intestinal and colonic mucosal epithelial cells were observed under microscope. No surgeons, nurses or anesthetists in our team were infected with SARS-CoV-2.

**Conclusions:**

It is meaningful and imperative to share our experience of protecting health care personnels from SARS-CoV-2 infection and providing references for optimizing treatment of cancer patients, at least for the operative intervention with absolute necessity or surgical emergency, during the outbreak of COVID-19.

## Background

The rapid spread of the novel coronavirus 2019-nCov, designated as severe acute respiratory syndrome coronavirus 2 (SARS-CoV-2) by the Coronavirus Study Group of the International Committee on Taxonomy of Viruses on February 12, 2020, has caused an ongoing outbreak of viral pneumonia and pandemic across the world since December 2019, which has been declared as a public health emergency of international concern by the World Health Organization [1, 2]. Patients suffered from severe or critical novel coronavirus pneumonia showed a higher mortality rate correlated with older age and increasing comorbidities (such as diabetes, cardiovascular and cerebrovascular diseases) [3]. Notably, patients with cancer are at increasing risk of severe infections and have a poorer prognosis due to the systemic immunosuppressive status caused by the malignancy [4]. Currently, several conservative therapeutic strategies have been recommended for colorectal cancer patients with coronavirus pneumonia, whereas surgery for patients with surgical indications are generally delayed [5]. On the other hand, it is suggested to withdraw cancer treatment such as chemotherapy, radiotherapy and targeted therapy for most patients infected with SARS-CoV-2 in case of deteriorating infection [6]. As a result, cancer patients suffered from a relatively high risk of tumor progression, making tumor therapeutic option a controversial point. Here, we first reported a colon cancer case infected with SARS-CoV-2 underwent radical resection of right colon without postoperative related complications and recovered from novel coronavirus pneumonia. Detailed pathological findings and our experience during the perioperative period were shared.

## Case presentation

**Fig. 1:**
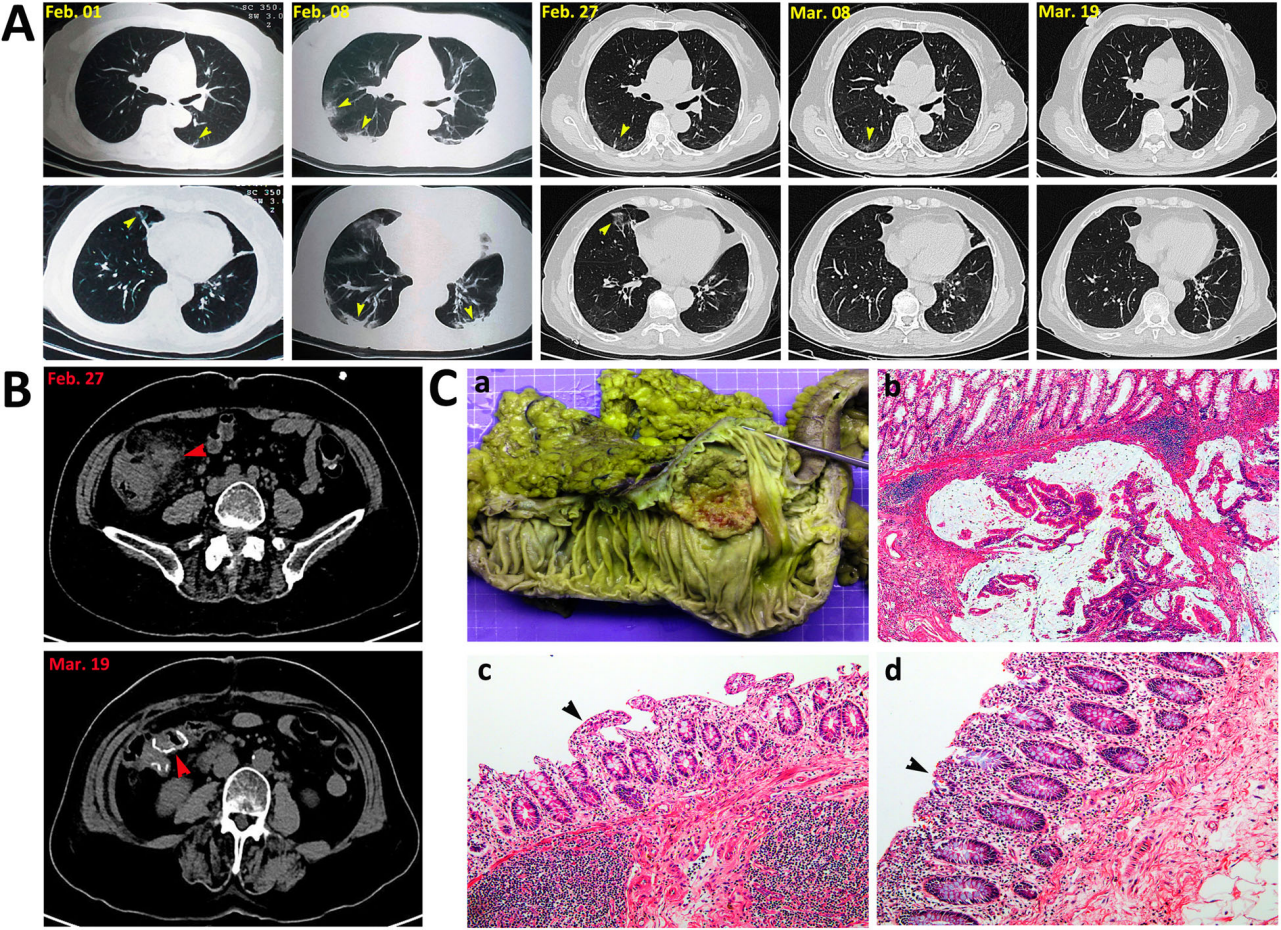
Representative images of chest CT scans and pathologic assessment. **a** Chest CT scans during the patient’s clinical course. The yellow arrows indicated patchy shadows in both lungs. **b** Preoperative CT scans on February 27 showed tumor extension, and postoperative CT scans indicated well-healed anastomosis on March 11, 2020. The red arrows indicate tumors or anastomosis, respectively. **c** Pathologic assessment of tissue specimen from colon cancer patient with SARS-CoV-2 infection. **a** The cut surface showed an ulcerative mass and necrosis in the center. **b** The histologic diagnosis of mucinous adenocarcinoma (H&E; 40×). **c** Degeneration, necrosis and slough of focal mucosal epithelial cells in terminal ileum, and **d** in colon of the ileocecal area (H&E;100×). The black arrows indicated histologic changes. CT = computed tomography; H&E = Hematoxylin & eosin staining

A 69-year old Chinese female patient presenting intractable intermittent fever, dry cough, chest tightness and right lower abdomen pain for 2 days was sent to our hospital for further novel coronavirus pneumonia and cancer treatments. On January 28, 2020, the patient developed a 38.4 °C fever, accompanied by fatigue and muscle soreness. This patient denied any medical, trauma or surgical history. Laboratory tests indicated slightly increased neutrophil percentage (74.0%) and decreased lymphocyte percentage (18.6%). A chest computed tomography (CT) scan on February 1 showed a few ground-glass opacities in the lower lobes of both lungs, suggesting suspicion for ‘infectious lesions of both lungs’ (Fig. 1a). Tests for influenza virus and other infectious agents were negative. However, the novel coronavirus nucleic acid test was not taken due to her mild symptoms and lack of nucleic acid detection kits. After treatment with Ceftriaxone and Abidol, her fever was not moderated yet. On February 8, a repeat CT scan showed ground-glass opacity lesions significantly increased compared with the previous one (Fig. 1a). Laboratory tests revealed remarkably decreased lymphocyte count (0.63 G/L), but normal neutrophil count (4.38 G/L). With adding methylprednisolone to the original treatment, patient’s fever gradually reduced to normal. Although the patient denied any contact with confirmed case in this period, a throat swab was obtained and the nucleic acid test for SARS-CoV-2 came back positively on February 20. However, she reappeared with fever on February 24, with a body temperature of up to 38.3 °C, and accompanied by dry cough, chest tightness and abdominal pain (especially in the right lower abdomen). Due to the poor curative effect in the local hospital, she was transferred to the infectious disease ward in our hospital on February 26. An abdomen CT scan indicated the tumorous lesion of the ascending colon with perforated and local wrapped changes on February 27 (Fig. 1b). The patient was diagnosed with ‘Severe novel coronavirus pneumonia’ and ‘Incomplete bowel obstruction: Colon cancer?’. Thereafter, the patient obtained a treatment for novel coronavirus pneumonia with Abidol and Moxifloxacin, as well as the corresponding symptomatic and nutritional support therapies. After above treatments, however, the patient still demonstrated intermittent fever while other accompanying symptoms were significantly relieved since February 29. Paradoxically, the follow-up results of nucleic acid retests for SARS-CoV-2 were negative on February 29, March 2 and March 10, respectively, with positive results of serum SARS-CoV-2 IgG antibodies on March 2, suggesting recovery from novel coronavirus pneumonia.

It is recommended to perform an aggressive surgical treatment for the patient, considering the fever caused by tumor, the risk of tumor progression and potential recurrences of abdominal pain, and even subsequent complete bowel obstruction which could make the general condition worse or be readmitted again. Furthermore, the patient did however present extreme motivation for surgery in an excellent general condition, optimistic mental state and well-nourished status despite the weight loss throughout the whole treatment period. Additionally, no other comorbidity factors contraindicated the operation. Based on carefully preoperative evaluation and preparation, our team performed an exploratory laparotomy for this patient via continuous epidural anesthesia, which took around 3.5 h, on March 11, 2020. Upon entering the abdominal cavity through a midline incision, we found that the tumor was approximately 6.0 cm × 5.0 cm, originating from the starting portion of ascending colon. The lesion was wrapped by omentum and surrounding tissues and accompanied by inflammatory adhesions to lateral abdominal wall and a portion of terminal ileum, which was consistent with CT scan results (Fig. 1b). No invasion to surrounding organs was found. Therefore, the right colectomy with lymph node dissection was performed. Five days after surgery, the patient could exhaust and defecate from the anus, suggesting relatively late recovery of bowel function. Until March 20, she gradually recovered without postoperative complications and was discharged on the 9th day after operation. Currently, the patient is being followed up closely as an outpatient.

The pathologic examination found a 5.0 cm × 5.0 cm ulcerative mass in the ileocecal area 1.0 cm from ileocecal valve, showing a gray white surface and necrosis in the center grossly. Microscopically, tumor invaded into the subserosal adipose tissue without lymph node metastasis, diagnosed as mucinous adenocarcinoma in the histologic diagnosis with pT3N0 (Fig. 1c). Of note, the significant degeneration, necrosis and slough of focal intestinal and colonic mucosal epithelial cells were observed in terminal ileum and ileocecal area surrounding the tumor, respectively (Fig. 1c).

## Discussion and conclusions

The rapidly ongoing outbreak of novel coronavirus disease 2019 (COVID-19), caused by SARS-CoV-2, has attracted not only Chinese but also worldwide concerns [1, 2]. As for cancer patients with COVID-19, whether to discontinue cancer therapy or defer surgical treatment remains controversial. To our knowledge, the case reported here represented the first colon cancer patient with confirmed COVID-19, who successfully underwent and benefited from the aggressive surgical treatment. During the period of manuscript preparation, no more surgery was performed for colon cancer patient infected with SARS-CoV-2, and thus clinic data on safety of surgical treatment and prognosis of such patients are sorely lacked. However, there are still positive implications and certain guiding significances for the treatment of colon cancer cases with emergency situation during the health care crisis.

Although the radical right colectomy is a standard surgical procedure for gastrointestinal surgeons, it becomes physically and technically challenging to perform the operation for colon cancer patient with confirmed SARS-CoV-2 infection in the specific period, because of its complexity which includes prolonged operation time and unclear vision, as well as discomfort and inconvenience caused by heavy personal protective equipments (PPEs). More importantly, several reasons give it less clinical priority to perform invasive diagnostic and surgical procedures, such as suddenness of the outbreak, high rate of transmission, vast patient volume in hospitals, and severe shortage of health care personnel [3], which indeed increases infection risk and mental stress for the medical team. In this case report, we successfully performed the operation for this patient with confirmed COVID-19. Everyone in our surgical team ensured to implement the standard tertiary protective measures against infectious diseases and applied active iodine on the surface of medical goggles to ensure a good surgical field, and then checked again with each other. Furthermore, we especially focused on responding to critical situations such as major bleeding, respiratory and cardiac arrest during the operation. Another two surgeons were on call all the time during surgery to deal with the shortage of surgical members due to significantly prolonged surgery time and excessive physical exertion of the surgeons. In order to minimize the production of iatrogenic aerosols, the anesthesiologist used continuous epidural anesthesia, but also prepared the equipment required for general anesthesia and rescue for timely responding to emergencies such as anesthesia accidents and respiratory distress during the surgery. We used electrocautery with suction device rather than used ultrasonic devices during the operation. Moreover, the surgery for the presented case was performed in a negative pressure operating room with laminar flow system, which could filter harmful gases and aerosols to the greatest extent. All these measures could effectively solve the problem of air and aerosols pollution in the operating room. Before entering the abdominal cavity, the operation needs to be gentle in case of body fluid splashing, which was also applicable during tissue dissection and surgical specimen removal.

A previous study has demonstrated that combining antiviral and anti-inflammatory treatments could simultaneously reduce viral infectivity, viral replication and the aberrant host inflammatory response [7]. Therefore, we recommended the preoperative treatment with combination of antiviral and anti-inflammatory medicine, so patients could significantly benefit from the processes of undergoing surgery and postoperative rehabilitation. Of note, there are some special patients that has been cured and discharged recently appeared in Wuhan, China, whose retesting results of nucleic acid for SARS-CoV-2 were positive again during outpatient follow-up and was re-admitted to the hospital. Therefore, it is prudent and important to regard all patients as potential infectious source during an epidemic season and cautiously practice “universal precaution” in perioperative period. Fortunately, thus far, no infection of surgeon, nurse or anesthetist in our team by SARS-CoV-2 occurred.

During the earlier phase of the COVID-19 outbreak, there were a significant number of health care providers infected with SARS-CoV-2 in many hospitals in Wuhan, and patients in the same room were cross-infected, due to exposure to unknown transmission sources. This dramatically increased the risk of SARS-CoV-2 infection for everyone in the outpatient clinics, especially cancer patients. On retrospective analysis of the patient’s medical course, the underlying cause of the first fever might be tumor-derived, and then were cross-infected with SARS-CoV-2 due to outpatient exposure. The SARS-CoV-2 infection and tumor lesion together caused a synergistic mechanism of activation and further aggravated the patient’s condition, which was partly clarified by the fever eventually reducing to normal after surgery. Pathologic findings revealed that the obvious degeneration, necrosis and slough of focal intestinal and colonic mucosal epithelial cells surrounding the tumor, which was consistent with the findings in a previous autopsy report [8]. Based on many clinical observing cases with diarrhea, it is probably one of evidences for gastrointestinal infection of SARS-CoV-2. However, these changes might also represent a nonspecific change with aging and could be considered as an important secondary change caused by tumor. More cases with sufficient controls are necessary to further clarify these pathological changes. Nevertheless, it is of great significance for clinicians to pay more attention to cancer patients and pathological changes of digestive tract organs during the outbreak of COVID-19. We believe that it is meaningful and imperative to share our experience to provide references for optimized treatment of cancer, at least for absolutely crucial surgical intervention or emergency surgery during COVID-19 pandemic.

## Data Availability

All data sets supporting the findings and inferences reported in this article are included within the article.

## Abbreviations

COVID-19: Novel coronavirus disease 2019
CT: Computed tomography
SARS-CoV-2: Severe acute respiratory syndrome coronavirus 2

## References

[1] Wang C, Horby PW, Hayden FG, Gao GF (2020). A novel coronavirus outbreak of global health concern. Lancet.

[2] Perlman S (2020). Another decade, Another Coronavirus. N Engl J Med.

[3] Tian S, Hu W, Niu L, Liu H, Xu H, Xiao SY. Pulmonary pathology of early phase 2019 novel coronavirus (COVID-19) pneumonia in two patients with lung cancer. J Thorac Oncol. 2020;15(5):700–4. 10.1016/j.jtho.2020.02.010 PMC7128866 32114094

[4] Liang W, Guan W, Chen R, Wang W, Li J, Xu K, Li C, Ai Q, Lu W, Liang H (2020). Cancer patients in SARS-CoV-2 infection: a nationwide analysis in China. Lancet Oncol.

[5] Yu GY, Lou Z, Zhang W (2020). Several suggestion of operation for colorectal cancer under the outbreak of Corona virus disease 19 in China. Zhonghua Wei Chang Wai Ke Za Zhi.

[6] Zhang H, Xie C, Huang Y. Treatment and outcome of a patient with lung cancer infected with severe acute respiratory syndrome coronavirus-2. J Thorac Oncol. 2020;15(5):e63–4. 10.1016/j.jtho.2020.02.025 PMC7128879 32147577

[7] Stebbing J, Phelan A, Griffin I, Tucker C, Oechsle O, Smith D, Richardson P. COVID-19: combining antiviral and anti-inflammatory treatments. Lancet Infect Dis. 2020;20(4):400–2. 10.1016/S1473-3099(20)30132-8 PMC7158903 32113509

[8] Yao XH, Li TY, He ZC, Ping YF, Liu HW, Yu SC, Mou HM, Wang LH, Zhang HR, Fu WJ (2020). A pathological report of three COVID-19 cases by minimally invasive autopsies. Zhonghua Bing Li Xue Za Zhi.

